# Influence of cultivar, irrigation, ripening stage, and annual variability on the oxidant/antioxidant systems of olives as determined by MDS-PTA

**DOI:** 10.1371/journal.pone.0215540

**Published:** 2019-04-18

**Authors:** Juan Antonio Sáinz, Inmaculada Garrido, Marcos Hernández, Alfonso Montaño, José Luis Llerena, Francisco Espinosa

**Affiliations:** 1 Department of Plant Biology, Ecology and Earth Sciences (Research Group FBCMP), Extremadura University, Badajoz, Spain; 2 European University of the Atlantic, Scientific and Technological Park of Cantabria, Santander, Spain; 3 Agri-Food Technological Center of Extremadura-CTAEX, Villafranco del Guadiana, Spain; Institute for Biological Research, SERBIA

## Abstract

The phenolic composition and content of olive fruit are some of the attributes that determine oil quality. This composition depends on the olive variety, the cultivation system, and the fruit's ripeness. This study considered two olive varieties (Manzanilla and Morisca), under two water regimes (irrigated and rainfed), harvested at three stages of maturation (S1, S2, and S3), over three consecutive campaigns (2011, 2012, and 2013). The accumulation of phenols in the fruit was found to depend only on the stage of ripeness, while the flavonoid and phenylpropanoid contents depended also on the variety and the water regime. Superoxide dismutase (SOD) activity was linked to O_2_^-^ production, which in turn depended on water regime, variety, and stage of maturation (this last being a process involving ROS). The peroxidase (POX) activity seemed only to depend on ripeness, while polyphenol oxidase (PPO) activity varied from year to year as well as presenting a strong ripeness dependence that was in clear coherence with the levels of phenolic compounds that the olives accumulate. All these relationships between the variables and the factors conform a dataset with the structure of a multidimensional array that is difficult to interpret using conventional techniques of statistical analysis. This work takes a novel approach (MultiDimensional Scaling associated with a Partial Triadic Analysis, MDS-PTA) to the analysis of this type of data structure which allows its correct interpretation. The analysis showed that the state of maturation of the olives is the most clearly discriminating factor, far more so than the cultivar, water regime, or year. Thus, the phenols and the total antioxidant activity (FRAP) showed strong clustering, being closely related in all three years studied. The oxidant and antioxidant activities showed a certain tendency to cluster, although in these cases the year also had an influence as a factor, indicating that these parameters depend more on external factors and less on ripeness.

## Introduction

The virgin oils obtained from olives (*Olea europaea*, L.) are an important source of lipids in the Mediterranean diet. In recent years, numerous studies have shown evidence for their nutritional and therapeutic properties [1]. This quality depends on various factors, among which are the cultivar, growing technique, state of maturity at the time of harvest, environmental factors, and extraction technique [2–4]. During the ripening of the fruit, biochemical processes take place affecting the content of sugars, phenols, etc., and these processes lead to changes in texture, firmness, and colour, which in turn determine the nutritional and organoleptic quality of the fruit [5]. Alterations during ripening occur at the membrane level, and end in a process of programmed cell death. Softening of the fruit involves processes of cellular oxidation and peroxidation [6].

The production of reactive oxygen species (ROS), which include O_2_^.-^ and H_2_O_2_, is a key part of plants' responses to stress and such physiological processes as ripening of the fruit [3,7–9]. Modulation of the levels of these compounds will depend on the balance between oxidant and antioxidant activities, a balance which controls the cellular redox state [5,10–12]. Respiratory burst oxidase homologues (Rboh) are responsible for the production of O_2_^.-^, oxidizing NADH. They play critical roles in the processes of plant growth and defence [13–15]. ROS must be strictly controlled to avoid triggering an oxidative stress [16]. Enzymatic antioxidant systems, such as SOD (superoxide dismutase), POXs (peroxidases), phenols, and carotenoids, are involved in this control [17–20]. SOD intervenes in the dismutation of O_2_^.-^ to the oxidant H_2_O_2_. Flavonoids and phenylpropanoids are oxidized by POXs and act as H_2_O_2_ scavengers. Polyphenol oxidase (PPO) activity is closely related to the aforementioned components of the antioxidant system. Indeed, these intervene in the hydroxylation of monophenols to diphenols, and in the oxidation of diphenols to quinones [21]. This process gives rise to the production of H_2_O_2_ which, together with phenols, can be substrates for POXs [22]. The interaction between POXs and PPO can determine the phenol content of olive oils in two ways. One is through the dependence of this content on the fruit's state of ripeness, and the other is through the release of phenols during milling [23]. POX activity is known to be related to the state of ripeness and to the secoiridoid content [3,24].

It has been demonstrated that lowered ROS levels delay ripening and senescence of the fruit, while raised H_2_O_2_ levels, as is the case during ripening, induce senescence [25,26]. There is also evidence that ROS play a key role in inactivating enzymes involved in ripening [27] and thus must be strictly controlled by enzymatic antioxidant systems. The balance between the production and the elimination of ROS is critical to maintaining the cellular redox equilibrium [10]. SOD, POXs, and PPO are related to this equilibrium in the fruit, and to protection against oxidative species [7,28].

One obstacle to better understanding these processes is that the corresponding biological, agronomic, and environmental data have a complex structure. Usually, they are collected together as sets (tables) of measurements and variables obtained under different experimental conditions or over different sampling periods, etc. Classical multivariate methods can deal with matrices (two-dimensional arrays), while this kind of data, with all of the data tables put together, usually results in a three-dimensional array [29,30].

The cultivar, irrigation, ripeness stage, and annual variability influence the enzymatic oxidant and antioxidant activities and such antioxidant compounds as phenols. A complete understanding of the effect of these factors would require an exhaustive experimental design, a multidisciplinary scientific approach, and an appropriate statistical method for the data's analysis.

The objective of the present study was to obtain an overview of the impact of the year, ripening stage, olive cultivar, and cultivation regime (irrigation or rainfed) on several biochemical parameters linked to oxidative stress of the fruit. To this end, we applied the technique of multidimensional scaling associated with a partial triadic analysis (MDS-PTA) as an alternative to a cluster analysis associated with partial triadic analysis (CA-PTA) proposed by other workers [29,31].

This novel approach has the advantage that it allows one to replace the vectorial coefficient analysis (vectorial correlation coefficient matrix) [32] by a visual exploration of figures generated. This type of scatter plots from MDS analysis is called common space, and thus identify which factor produces most homogeneity.

## Material and methods

### Olive sampling and sample preparation

The experiment was carried out in Extremadura (mediterranean climate) in two olive groves (*Olea europaea*, L), one of cv. Manzanilla de Sevilla (MAN), and the other of cv. Morisca (MOR). The groves' location is at Ribera del Fresno (Badajoz, Spain). The trees were of 25 years old, and both groves are maintained under conditions of no-tillage, post-emergence herbicide weed control, and traditional pruning. The climatic conditions are summarized in Table 1.

**Table 1:** Mean temperatures (máximum and minimum) and monthly rainfall, evapotranspiration (ETo) and irrigation* during 2011, 2012 and 2013 in the location estudied.

	2011	2012	2013	2011	2012	2013	2011	2012	2013	2011	2012	2013	2011	2012	2013
	Max temp (°C)			Min temp (°C)			Rainfall (mm)			Eto (mm)			Irrigation (m^3^ ha^-1^)		
January	12.26	14.23	13.06	4.17	1.26	4.57	33.60	15.64	40.60	31.12	35.64	32.46			
February	15.85	14.52	13.10	3.74	-1.04	2.51	27.80	1.19	77.00	53.22	63.51	46.31			
March	16.63	20.27	15.07	6.30	5.44	6.97	57.34	2.18	104.20	75.99	108.60	66.19			
April	23.61	17.13	20.19	10.94	6.86	7.55	37.82	53.86	23.60	121.95	99.10	114.47	246.40	327.65	236.59
May	26.88	27.20	23.49	13.70	12.87	9.39	23.96	20.99	16.40	153.94	171.95	154.34	387.23	446.11	568.58
June	30.83	31.27	29.09	14.70	15.53	14.23	6.73	0.00	10.80	197.24	205.86	182.57	495.89	554.19	400.83
July	32.99	33.85	34.72	16.66	16.41	18.20	0.00	0.00	0.80	223.70	225.34	216.96	621.23	615.71	568.59
August	32.90	34.37	34.83	17.47	17.30	18.21	21.38	0.00	1.60	185.62	197.33	195.12	611.68	613.40	408.43
September	30.29	29.00	30.40	15.07	15.63	16.47	11.29	31.50	12.00	132.39	127.17	138.23	401.92	402.56	346.19
October	26.56	22.23	23.57	12.08	11.16	11.70	59.80	84.60	68.20	99.82	77.51	79.52	224.00	165.76	164.27
November	16.95	15.68	15.40	7.73	7.57	4.03	64.35	148.20	4.60	45.21	36.95	39.83			
December	13.28	13.71	14.51	3.13	4.59	2.75	10.49	32.60	44.60	28.60	27.17	36.34			
Mean temp	23.25	22.78	22.28	10.47	9.46	9.71									
Total rainfall							354.56	390.76	404.40						
Total ETo										1348.80	1376.13	1302.34			

* Only in FI treatment

The olive samples were harvested during the months of September, November, and December, from 2011 to 2013. The crops were grown under two types of water regime–irrigation in accordance with the levels of crop evapotranspiration (FI), and rainfed (NI). In the irrigated grove (FI), water is applied in accordance with the trees' theoretical requirements (calculated using Eto and Kc -crop coefficient-) ussing to the recommendation from Junta de Extremadura. The irrigation period was extended from 1^st^ april to 31 october (Table 1). The soil type of both plots is sandy clay loam.

Olives were randomly harvested in the same stage of coloration, from around the canopy of ten tree of each variety and experimental condition, at a heigh of 1.5 m. The recollection at three different stages of ripening: green (S1, 0–1), veraison (S2, 2–3), and black or mature (S3, >3) were carried out in september (second week), november (second week) and december (first week), respectively, in accordance with Uceda and Frias [33]. Fruits were sampled between 9 and 10 h AM and immediately frozen in liquid nitrogen until the biochemical analysis. The fruit yield (kg ha^-1^) and oil concentration (% dry weight) were determined by near-infrared (NIR; Olivesan equipe FOSS), and total oil production per ha calculated.

### Determination of the dry weight/fresh weight ratio, and the soluble amino acid and protein contents

For the determination of the dry weight/fresh weight (DW/FW) ratio, the olive pulp was first weighed fresh and then oven-dried at 90°C for 24 h, after which time the dry weight was measured.

The olive pulp was homogenized in 50 mM sodium phosphate buffer, pH 7.0 (0.2 g mL^-1^), then filtered through muslin, centrifuged at 12360 *g* for 15 min, and the supernatant used for the protein and amino acid assays. For the protein assay [34], aliquots of 50 μL or 100 μL of supernatant were mixed with 200 μL of Bradford reagent, and completed to 1 mL with distilled water. After 30 min incubation at room temperature, the absorbance was measured at 595 nm. The results are expressed as mg of proteins g^-1^ FW against an albumin standard curve. For the amino acid assay [35], aliquots of 100 μL of supernatant were mixed with 1.5 mL of ninhydrin reagent (80 g of stannous chloride dissolved in 50 mL of 200 mM sodium citrate buffer, pH 5.0, plus 2 g of ninhydrin dissolved in 50 mL of ethylene glycol). Following 20 min incubation at 100°C, the mixture was cooled, 8 mL of 50% propanol was added, the result was left at room temperature for 30 min, and then the absorbance was measured at 570 nm. The results are expressed as mg of amino acids g^-1^ FW against a glycine standard curve.

### Total phenols, flavonoids, and phenylpropanoid glycosides

Phenols, flavonoids, and phenylpropanoid glycosides (PPGs) were assayed colorimetrically. First, the olives were homogenized with methanol, chloroform, and 1% NaCl (1:1:0.5). The homogenate was filtered and centrifuged at 3200 *g* for 10 min and the methanolic phase separated, where the phenolic compounds were determined. Total phenols (expressed as μg caffeic acid g^-1^ FW) were determined at 765 nm with Folin-Ciocalteu reagent in accordance with Singleton et al. [36]. Total flavonoids (expressed as μg rutin^-1^ FW) were determined in accordance with Kim et al. [37], calculating the content on the basis of the rutin standard curve. The PPG levels (expressed as μg verbascoside g^-1^ FW) were determined at 525 nm based on estimating an *o*-dihydroxycinnamic derivative using Arnow reagent as described in Gálvez et al. [38], calculating the content on the basis of the 3,4-dihydroxyphenylalanine standard curve.

### Enzymatic oxidant/antioxidant activities

Enzymatic activities were determined on an extract of the raw olives. For the PPO activity, the olive samples (0.15 g mL^-1^) were homogenized at 4°C in 100 mM phosphate buffer, pH 7.0, 1% PVPP. The homogenate was filtered and centrifuged at 12000 *g* for 15 min at 4°C. The pellet was discarded, and the supernatant filtered, collected, and immediately used for assay. For the other enzymes, the olives (2 g mL^-1^) were homogenized at 4°C in 50 mM phosphate buffer, pH 6.0. The homogenate was filtered and centrifuged at 39000 *g* for 30 min at 4°C. The pellet was discarded, and the supernatant filtered for the assays and protein content was determined [34].

NADH oxidation was measured by the fall in A_340 nm_ [39] (ε = 6.3 mM^-1^ cm^-1^), and expressed as nmoles NADH_ox_ min^-1^ mg^-1^ prot. The reaction mixture consisted of the enzyme extract and 300 μM NADH in 50 mM phosphate buffer pH 6.0.

The O_2_^.-^ generating activity was measured spectrophotometrically from the oxidation of epinephrine to adrenochrome at A_480 nm_ (ε = 4.020 mM^-1^ cm^-1^) [40,41]. The reaction mixture consisted of the enzyme extract and 1 mM epinephrine in 25 mM, pH 5.0 acetate buffer. The result is expressed as nmol adrenochrome min^-1^ mg^-1^ prot.

The SOD (EC 1.15.1.1) activity was determined from the absorbance at 560 nm of the enzyme extract in 50 mM pH 7.8 phosphate buffer, 0.1 mM EDTA, 1.3 μM riboflavin, 13 mM methionine, and 63 μM NBT [42]. A unit of SOD is defined as the amount of enzyme required to cause 50% inhibition of NBT reduction.

The POX (EC 1.11.1.7) activity was measured at 590 nm (ε = 47.6 mM^-1^ cm^-1^) [43], with the reaction medium consisting of the enzyme extract, 3.3 mM DMAB, and 66.6 μM MBTH in 50 mM phosphate buffer, pH 6.0. This activity is expressed as nmol DMAB-MBTH (indamine dye) min^-1^ mg^-1^ prot at 25°C, pH 6.0.

The PPO (EC 1.14.18.1) activity was determined from the absorbance at 390 nm and 30°C of a reaction medium consisting of the enzyme extract, 100 mM phosphate buffer, Triton X-100, and 30 μM caffeic acid [44]. A unit of PPO is defined as the amount of enzyme required to cause a decrease in absorption of 0.001 units min^-1^.

### Total antioxidant activity assay (FRAP)

Determination of the ferric reducing ability of plasma (FRAP) was performed in accordance with Rios et al. [45]. Olive samples were homogenized with methanol (0.10 g mL^-1^). The homogenate was filtered and centrifuged at 10000 *g* for 2 min at 4°C. Aliquots of 10 μL homogenate were mixed with 1500 μL FRAP reagent, maintained at room temperature for 5 min, and then measured at A_593nm_. Calibration was done against a standard curve using freshly prepared ferrous ammonium sulfate, and the concentration was expressed as μg of ferrous sulfate g^-1^ FW.

### Data analysis. Multidimensional scaling associated with partial triadic analysis (MDS-PTA)

Partial triadic analysis (PTA) belongs to the STATIS family of exploratory tools for three-way data analysis. It is based on the logic of PCA (principal component analysis), and, as input, takes data that can be arranged in a three-dimensional array [46–48]. Its main purpose is to compare matrices of data that were obtained under different experimental conditions [30].

In PTA, all the data matrices must have the same dimensions, but its advantage is that it works with the original data instead of working with operators, so the results can be interpreted in a direct manner [49,50]. It comprises three stages: inter-structure, compromise, and intra-structure [29,32].

In the specific case of our proposal, which is suggested to nominate as MDS-PTA, the first stage (the inter-structure analysis) is replaced by a visual analysis of the common space (the low-dimensional space into which the individuals are projected in terms of their similarity). The aim is to identify which factor produces the greatest homogeneity. In the present work, after carrying out this first stage, for the following compromise and intra-structure stages, we proceeded in a similar way to that described by Mendes et al. [49], Gourdol et al. [29] and Darwiche-Criado et al. [31].

In order to obtain a common space, a proximity matrix is required to perform MDS. This is a collection of estimates of the similarity of each pair of samples in the set of biochemical constituents. The proximity was taken to be the simple Euclidean distance which is independent of the choice of origin and the orientation of the coordinate axes [51].

The proximity matrix was analysed using the PROXSCAL (PROXimitySCALing) algorithm. The configurations produced by PROXSCAL tend to give a more even spread of the object points throughout the low-dimensional common space (the MDS diagram) [52]. A scree plot and Shepard diagram were used to evaluate how well a particular configuration reproduced the observed proximity matrix. In a scree plot, the stress (a measure of the disagreement between the estimated distances and the input proximities) is plotted against the number of dimensions. An "elbow" in this plot indicates the best number of dimensions. A Shepard diagram is a scatter plot displaying the relationship between the proximities and the distances in which less spread implies a better fit [53].

Although the samples were defined by four qualitative factors (year, ripening stage, cultivar, and irrigation), only the year and ripening stage were finally used to determine the third axis of the data set since these two factors have a temporal character.

All the statistical analyses were applied to standardized data using the IBM SPSS vn 21.0 statistics software package (SPSS Inc., Chicago, IL, USA).

## Results and discussion

### Fruit and oil yield

The production in the two cultivars shows a positive effect produced by the input of irrigation (Table 2). In the three years which were studied, both cultivars increased the production as an answer to irrigation. The cv. Manzanilla shows a similar effect as that described by Sofo et al. [54] regarding the ON (2011 and 2013) and the OFF (2012) years. In regard to the oil concentration (expressed on a dry weight basis), in the three years oscillates between 40.0% and 50.6% for MOR, and between 40.9% and 48.0% for MAN. The average oil concentration in NI and FI conditions is similar, except for MAN in FI (slightly smaller). These results indicate that irrigation does not significantly affect the total concentration of oil. A slight tendency towards the decrease of this concentration in conditions of input of irrigation can be observed, only significant in 2011 for the cv MAN. Total oil yield does increase in irrigation conditions, but in this case it is due to the increase of olive production, which increases considerably in both varieties. These results are in tune with those obtained by Cirilli et al. [55] and Ahumada-Orellana et al. [56], in cultivars Frantoio and Arbequina respectively, where it is observed how the input of different levels of irrigation increases both the production and the oil yield, but not altered the total oil content. The effect of irrigation in total oil content is not seen, attributing the oil yield to the maturity index rather than the water supply.

**Table 2:** Fruit yield, oil concentration and oil yield of olive trees for each cultivar, treatment and year. To compare the means of the variables, a ANOVA and post-hoc test was applied. Significant differences were taken into account when p-value < 0.05.

Cv	Irrigation	2011	2012	2013	2011	2012	2013	2011	2012	2013
		Fruit yield (kg ha^-1^)			Oil concentration (% dry weight)			Oil yield (kg ha^-1^)		
MOR	NI	4686.3^c1^	3448.0^b1^	1905.0^a1^	40.0^a1^	41.4^a^	50.6^b^	1874.5^b1^	1427.5^b1^	963.9^a1^
	FI	6952.4^c3^	5390.7^b2^	3602.2^a2^	44.6^12^	42.7	47.2	3100.7^c3^	2301.8^b2^	1700.2^a2^
MAN	NI	5886.4^c2^	4991.3^b3^	4953.0^a3^	46.1^2^	48.0	45.9	2713.6^2^	2395.8^2^	2273.4^3^
	FI	7758.5^c4^	5365.5^a2^	7002.8^b4^	40.9^a1^	47.1^b^	44.1^ab^	3173.2^b3^	2527.1^a2^	3088.2^b4^

Significant differences among years (raw) are indicated in letter. Significant differences in the same group by years (fruit and oil yield, and oil concentration) are indicated in number.

### DW/FW, amino acid and protein contents, total phenol content, oxidant and antioxidant activities, and total antioxidant activity

The DW/FW ratio (Table 3, and S1 Table) remained practically constant throughout the ripening stages of the olives studied. No alterations of this ratio were observed that were dependent on either the variety or the year–only a certain influence of irrigation, with a lower DW/FW ratio in the irrigated olives, as was to be expected from the increased water content of these olives. With respect to the total content of soluble amino acids and proteins, these two components showed similar behaviour, as expected given their close relationship. There was an increase with ripening, with the highest levels at S3, and the Morisca cultivar had higher values. This result contrasts with that described by Zamora et al. [57] for cv. Arbequina and Picual, who observed no significant increases in protein content during ripening, although in the cv. Arbequina there did occur slight increases in these components. To the best of our understanding however, the data described by those workers for these two varieties should be taken with some precaution since that study only considered data from a single year, whereas our case corresponds to 3 years. The potential influence of external factors means that the behaviour observed for an isolated year might be very different from that observed in another. Thus, those workers' data would be very similar to our observations in 2013 in these two varieties, and very similar to our observations for cv. Morisca in 2012. It is important to stress that in the case of field experiments in olive groves, the evolution of the parameters can vary greatly due to their dependence on very many environmental factors that are difficult to control and that determine the physiological characteristics of this crop. Thus, Ebrahimzadeh et al. [58] observed in the cv. Zard but in very different locations the existence of what they termed "on" and "off" years between which the olives' total protein levels varied very considerably. They also observed major differences in those levels depending on the geographical location, again demonstrating the strong influence of environmental conditions as a whole, including the edaphic and cultivation conditions. Those workers also found the total protein content to increase with ripening, as in our case. Similar data were obtained by Ortega-García et al. [59] in cv. Picual, with a clear increase in the olives' protein content with ripening. At least in our case, the variety also affects the protein content. Thus, cv. Morisca had higher levels of both soluble amino acids and proteins than cv. Manzanilla, regardless of the stage of ripening. Whether the cultivation regime was rainfed or irrigated had no differential effect on the contents of the two types of compound. This variety-dependent behaviour has been put forward as a possible new varietal marker, both using the pulp content and subsequently using the oils that are obtained [60]. In sum therefore, we can say that the olives' protein content and its evolution with ripening are highly variable, with a dependence not just on the variety but on a large number of external factors.

**Table 3:** Values of means of variables obtained from S1 Table. A t-Student test were applied on olive cultivar and irrigation cases while ANOVA with Duncan´s post-hoc test was applied on ripening stage (IM).

Year	Parameter	IM			Cultivar		Irrigation	
		S1	S2	S3	MOR	MAN	NI	FI
	DW/FW	0.31±0.04^a^	0.29±0.01^a^	0.36±0.01^b^	0.31±0.04	0.33±0.03	0.32±0.04	0.32±0.04
	Soluble amino acids	2881.39±1302.18^a^	2143.42±1373.52^a^	5997.94±1205.93^b^	4407.07±1990.09	2941.44±2114.21	4057.55±2235.33	3290.95±2090.42
	Total proteins	0.8±0.22^a^	1.39±0.51^b^	3.39±0.28^c^	2.07±1.24	1.65±1.24	1.93±1.23	1.79±1.29
	Total phenols	1728.66±21.34^a^	1914.04±109.03^b^	2219.83±5.34^c^	1933.56±243.16	1974.79±213.8	1976.51±218.02	1931.84±239.01
	Total flavonoids	4329.78±350.09^a^	5136.93±726.72^b^	6965.51±64.86^c^	5202.72±1414.3	5752.09±1062.8	5360.79±1369.48	5594.03±1184.39
2011	Total PPGs	7201.57±428.02^a^	7574.32±1130.14^a^	9535.05±291.91^b^	8000.37±1559.27	8206.93±992.34	8275.19±1049.53	7932.1±1506.49
	NADH oxidation	700.52±351.12	586.98±95.40	410.33±110.63	459.73±92.89	672.15±292.05	564.57±262.15	567.31±228.62
	O_2_^.-^ production	261.77±100.97	226.22±96.93	193.69±93.53	201.06±63.09	253.39±115.04	243.36±112.58	211.1±74.55
	SOD	104.09±56.86	57.77±8.38	76.04±27.98	55.51±10.01^a^	103.09±43.18^b^	75.37±33.99	83.23±46.15
	POX	129.49±68.02	116.53±32.91	72.44±20.56	82.93±23.46	129.38±57.14	106.23±58.77	106.07±40.70
	PPO	620.78±97.54^b^	549.55±147.42^ab^	395±60.50^a^	575.88±159.94	467.67±98.26	505.37±161.44	538.19±124.7
	FRAP	21.53±0.27^a^	26.65±0.27^b^	27.91±0.11^c^	25.36±3.14	25.38±2.91	25.36±3.14	25.37±2.91
	DW/FW	0.33±0.04	0.30±0.04	0.30±0.03	0.32±0.04	0.30±0.03	0.33±0.03^b^	0.28±0.02^a^
	Soluble amino acids	2129.13±372.13	2871.67±1124.23	4068.86±1845.63	3709.01±1723.58	2337.43±564.23	2700.51±1412.64	3345.92±1473.89
	Total proteins	1.27±0.53^a^	1.69±0.65^ab^	2.3±0.28^b^	2.16±0.39^b^	1.34±0.59^a^	1.74±0.66	1.76±0.67
	Total phenols	3364.66±341.11^b^	2378.22±459.13^a^	4275.16±175.37^c^	3456.69±852.88	3222.01±945.74	3431.36±875.33	3247.34±931.85
	Total flavonoids	8036.74±1173.19^b^	5822.83±825.84^a^	8864.37±657.16^b^	7830.58±1825.51	7318.72±1400.4	8226.61±1511.64	6922.69±1464.79
2012	Total PPGs	13707.22±1702.28^b^	10188.89±1712.84^a^	12530.53±1539.8^ab^	12380.74±2671.44	11903.68±1667.58	13379.02±1785.77^b^	10905.4±1787.10^a^
	NADH oxidation	525.17±178.54	1303.38±523.29	986.75±238.28	967.24±605.10	909.63±307.24	1036.87±551.93	840±366.95
	O_2_^.-^ production	296.75±65.86^a^	646.79±289.96^b^	458.61±63.37^ab^	535.55±292.27	399.22±89.37	501.64±286.98	433.14±139.25
	SOD	82.14±46.23	155.97±61.76	126.72±33.36	82.12±32.45^a^	161.1±40.69^b^	126.27±59.56	116.95±53.39
	POX	104.32±41.49^a^	206.01±60.77^b^	114.99±27.87^a^	146.33±88.73	137.21±28.09	140.02±68.81	143.52±63.01
	PPO	364.39±90.72	309.75±82.51	401.67±79.85	383.53±67.15	333.67±101.33	382.1±64.82	335.1±103.64
	FRAP	114.93±5.67	107.63±18.88	127.95±9.66	111.07±11.42	122.61±15.77	122.19±15.59	111.49±12.13
	DW/FW	0.28±0.03	0.29±0.04	0.3±0.02	0.29±0.02	0.28±0.04	0.31±0.01^b^	0.27±0.03^a^
	Soluble amino acids	1534.27±360	1850.05±747.9	2320.39±409.15	2252.1±436.51^b^	1551.04±527.98^a^	1903.97±428.98	1899.17±761.68
	Total proteins	1.01±0.47^a^	1.76±0.45^b^	1.97±0.10^b^	1.84±0.34	1.32±0.62	1.57±0.52	1.59±0.63
	Total phenols	3450.59±404.68	2932.24±300.09	3441.97±245.84	3398.78±389.4	3151.08±375.58	3411.8±437.74	3138.06±304.84
	Total flavonoids	7236.66±1511.28	6067.16±1191.29	7025.39±721.62	7291.89±1189.2	6260.91±1050.64	7297.63±1284.54	6255.17±923.97
2013	Total PPGs	12860.89±2128.31	11296.54±1863.61	11465.14±1985.75	12599.61±1998.58	11148.77±1757.82	12888.83±1832.84	10859.54±1586.54
	NADH oxidation	361.13±52.54^a^	1018.83±314.71^b^	683.53±418.01^ab^	706.32±465	669.34±349.18	689.15±488.34	686.51±317.01
	O_2_^.-^ production	245.05±42.66	325.61±44.68	265.62±114.75	295.25±95.55	262.26±56.67	278.54±78.72	278.98±82.44
	SOD	84.05±39.23^a^	149.73±43.12^ab^	179.91±41.36^b^	104.85±45.92	170.94±46.68	142.49±65.43	133.29±50.77
	POX	57.25±14.06^a^	147.01±29.95^b^	71.42±50.02^a^	85.39±51.98	98.39±55.51	85.6±60.06	98.19±46.71
	PPO	365.76±116.23^ab^	232.17±42.32^a^	391.27±32.48^b^	364.67±89.56	294.79±102.69	334.75±106.69	324.71±100.27
	FRAP	98.47±13.10	83.69±23.55	107.01±20.23	91.93±18.5	100.84±22.67	107.25±17.71^b^	85.52±17.51^a^

For each parameter values with different letters are significantly different (*p* < 0.05). Soluble amino acids and total proteins expressed as mg g^-1^ FW; NADH oxidation, O_2_^.-^ production and POX activity expressed as nmoles min^-1^ mg^-1^ protein; SOD and PPO activities was expressed as U mg^-1^ protein; total phenols, total flavonoids, PPGS and total FRAP expressed as μg g^-1^ FW.

The total content of phenols, flavonoids, and PPGs showed a similar behaviour in the three years studied (Table 3, and S1 Table). In 2011, these values were significantly different from those obtained in 2012 and 2013 (which were similar to each other). Such year-to-year fluctuations have also been described for cv. Cheniali and cv. Arbosana [54]. All the types of phenols assayed increased throughout ripening, with the greatest values corresponding to the mature state (S3). The total phenols dependent only on the ripeness stage. The total flavonoid and PPG contents showed a dependence on cultivar, irrigation, and ripeness stage. The cv. Morisca presented greater flavonoid and PPG contents than cv. Manzanilla, and the rainfed olives of both cultivars had greater contents of these compounds. Nonetheless, the general trend shown by both cultivars was a decrease with ripening up to stage S2, with a subsequent increase to S3. The content and evolution of these compounds depends to a large extent on the variety studied. Thus, Ortega-García et al. [59] report that in rainfed cv. Picual there were slight fluctuations in the total phenolic content as ripening proceeded, while Ortega-García and Peragón [61] in cv. Picual, Verdial, Arbequina, and Frantoio together with Cerretani et al. [62] and Morelló et al. [63] (cv. Nostrana di Brisighella and Ghiacciolo, and Arbequina, Farga, and Morrut, respectively) report decreases in the levels of phenolics with ripening. This last work, however, also found that, while the flavonoids presented an overall decreasing trend with ripening, the three varieties studied presented major differences in the total content and the fluctuations of those flavonoids. Finally, Sobhy El Sohaimy et al. [64] in cv. Manzanilla and Kalamata describe declines, although very slight, in both total phenolics and flavonoids with ripening. Together, these results show the great variability depending on the stage of ripeness, the variety, and, above all, the specific conditions affecting the year of study since this last factor can contribute to the development of stress responses, a process in which compounds of this type are involved in eliminating H_2_O_2_ and in acting as detoxifying agents.

The NADH oxidation capacity showed strong variations by year and by cultivar in 2011, while irrigation had no clear effect (Table 3). Neither was there any definite pattern of behaviour with respect to the ripeness stage. There were fluctuations in some cases, but in others the values remained practically constant from S2 to S3. Taking the 3 years' NADH oxidation values together, one observes that, for the rainfed crops, the greatest NADH oxidation corresponded to S2, with a greater incidence in cv. Morisca than cv. Manzanilla. In Morisca, there was an increase from S1 to S2 in all cases, followed by a decrease at S3. The case was similar for cv. Manzanilla except in 2011. On the contrary, under irrigation, in both cultivars there were either increases in oxidation with ripening or no change between S2 and S3. The formation of O_2_^.-^ was strongly related to this oxidation activity. In this case, there were alterations similar to those described above, with no clear trend or effect of any of the factors studied regarding the determination of their evolution. The O_2_^.-^ production levels were similar except for the cv. Morisca in 2012 which were much higher. In the first case, there were no differences due either to the cultivar or to irrigation. The ripeness stage seems to have some influence. Thus, for the Morisca cultivar under irrigation, there was an increase with ripeness, whereas for the rainfed olives, and probably in response to it, the initial increase was followed by a decrease at S3. The cv. Manzanilla showed either slight falls with ripening or stable situations. In 2012, there was greater production in cv. Morisca, with a strong incidence of the rainfed and ripeness conditions, with the greatest values being reached at S2. The production of O_2_^.-^ over the whole 3 years had a similar evolution to the NADH oxidation. Rboh are responsible for O_2_^.-^ production, with NADH as electron donor. The production of O_2_^.-^ and H_2_O_2_ in the fruit increased in the middle and late stages of ripening (Table 3, and S1 Table), with a subsequent decrease in the final phase of ripening. These results are similar to those obtained elsewhere [7,65,66].

The control of O_2_^.-^ levels and the maintenance of cellular redox homeostasis involves the action of such antioxidant systems as SOD and POXs, and showed the relation to ROS and ripening. The presence of SOD and other antioxidant enzymes in olives was demonstrated by López-Huertas and del Rio [67] in cv. Picual. The part they play is especially important during ripening since, by eliminating ROS, they protect polyphenols from oxidation. The SOD activity that we determined shows a behaviour that is clearly dependent on the cultivar (S1 Table): cv. Manzanilla had much higher (×2) levels than cv. Morisca. In both cultivars, there was a non-significant increase in response to rainfed cultivation. There were fluctuations throughout ripening, without any clearly defined pattern. Such ripeness-dependent variations have previously been reported in grapes [9] for which, as in our case, there were differences according to cultivar. These activities increased during ripening, but this may have been due to the activation of these systems in defence against stress, which also may explain the different behaviours according to cultivar, irrigation, and year. For the whole three years together, the rainfed crop showed an increase with ripening from S1 to S2, followed by a decline to S3, but in the irrigated crop this final decline was less marked or non-existent. Other workers have found a similar evolution of this activity throughout ripening in plum and peach [65,66]. These fluctuations may be related to increases in O_2_^.-^ during ripening, on which will depend a greater or lesser SOD activity [9]. The effect is more evident when the data of the two activities are taken together for all 3 years–an increase in the amounts of both O_2_^.-^ and SOD activity. This behaviour is very similar to that described in cv. Gordal and Manzanilla by Hornero-Méndez et al. [28] who attribute the greater activity they observed in cv. Gordal to this cultivar's greater susceptibility to oxidative stress, and hence greater SOD activity in defence against the development of oxidative damage. In our case, it was cv. Morisca that produced the greater amount of O_2_^.-^, but with lower levels of SOD activity. This seems to indicate the participation of some other system of defence against oxidative damage, i.e., greater amounts of flavonoids and PPGs and a lower sensitivity to this damage in this cultivar. The cv. Manzanilla presented lower levels of O_2_^.-^, but considerably greater SOD activity. This could mean that this cultivar is more sensitive to this type of stress, and requires this stress to be rapidly and effectively controlled.

The POX activity presented a similar behaviour in all three years. While there were no significant differences with respect to irrigation, there were with respect to cultivar and stage of ripening (Table 3, and S1 Table). This activity declined throughout ripening in cv. Manzanilla, but in Morisca it rose in the initial phases (from S1 to S2) and then either declined or, in some cases remained unchanged by S3. In Manzanilla, the maximum values of this activity showed little fluctuation, being fairly constant in the different years; the values were quite similar in 2011 and 2012, decreasing with ripeness, but in 2013 their evolution was similar to that of Morisca in 2011 and 2012, with a strong increase at S2 followed by a decline. Hachicha Hbaieb et al. [4] observed slight fluctuations with ripening in cv. Chetoui, but no variation in Arbequina, as also had García-Rodríguez et al. [23] in cv. Arbequina and Picual, although in the latter there was a slight decrease in the fully ripe state with respect to intermediate stages of ripening. To the contrary, Cirilli et al. [55] found a decline in this activity with ripening in cv. Frantoio, with just a slight increase in the final stages, although there were greater fluctuations under irrigation. The results obtained in our varieties are different from those just described, confirming the great variability of this activity according to variety and year. In our case, the behaviour of cv. Manzanilla (2012 and 2013) was very similar to that described for Frantoio, especially in the rainfed regime, with strong declines in activity from S1 to S3. Both SOD and POX were highly variable between years, with the cv. Morisca SOD activity at S2 being much greater in 2012 and 2013 (by a factor of 2) than in 2011. The cv. Manzanilla also presented these fluctuations, showing a similar evolution with ripening to that of Morisca. Such year-to-year fluctuations have also been described by Sofo et al. [54] for other olive cultivars. With regard to the POX activity, in Morisca the values at S2 in 2012 were much higher (×1.8) than in 2011 and 2013. In Manzanilla, there was little fluctuation in this activity, with the levels being more constant, except in 2013 where the behavior and the values in each madurtiy stage show a similar evolution to Morisca.

With respect to the PPO activity (Table 3, and S1 Table), there were no differences between irrigation and rainfed cultivation, but there were clear differences between years. In all cases, the cv. Morisca presented a greater PPO activity. The evolution of PPO with ripening also differed according to the cultivar. For Morisca, the PPO declined from the beginning of ripening until its completion at S3, a result that is coherent with what has been described for cv. Gordal and Picual [28]. For cv. Manzanilla, PPO only declined with ripening in 2011 when the activity levels were very high. In the other two years, the PPO values were similar throughout ripening with just a slight final rise. This varietal dependence of the behaviour has also been observed in cv. Picual and Arbequina [23]. Hachicha Hbaieb et al. [3,4] describe fluctuations (cv. Chétoui) or declines (cv. Arbequina) with ripening. The different PPO activities that were observed coincide with the evolution of the amount of phenols determined for the two treatments. On the other hand, Ortega-García et al. [59] with cv. Picual shows an increase in PPO activity with the ripening, as does Cirilli et al. [55] in the cv. Frantoio, who also observe a greater increase in irrigated conditions. The lower PPO activity at the S3 allows the levels of low oxidized phenols to be fulfilled, what is key to the quality of the oil olive virgin extracted from this olive fruits. The SOD, POX, and PPO activities are involved in the processes controlling the redox balance by stabilizing the levels of ROS that are produced during ripening and in response to environmental stresses. The SOD and POX activities evolve in a closely related manner, with fluctuations that are similar to each other, and in turn their evolution is related to the production of ROS. This reflects their conjoint action in controlling ROS. Furthermore, PPO and POX not only intervene in this process but also in the oxidation and modification of the phenolics profile of the olives during their ripening, especially of the phenolic glycosides [23]. As a result, the two activities are modulated throughout ripening. The great variability observed in the 2 varieties, during the 3 years of study, in 3 stages of ripening, and under 2 cultivation regimes, may be due to the conjoint influence of environmental factors and maturation, with this latter being the factor that discriminates most strongly. These activities also in part depend on the variety [28]–the MAN variety presents higher levels of SOD activity than MOR, but, in contrast, MOR shows higher levels of POX and PPO activities. The influence of environmental factors, such as the cultivation regime, is reflected in the higher SOD and POX activities under rainfed than under irrigated conditions, with this factor strongly affecting the evolution of these activities during ripening. The PPO activity, however, does not show this influence, but is more dependent on the state of ripening.

The total antioxidant capacity was lower in 2011 than in the other two years, but it was influenced by neither the cultivar nor irrigation (Table 3, and S1 Table), and stayed fairly constant in all cases within any given year. The phenol content may determine the total antioxidant capacity. In chickpeas (*Cicer arietinum*), total antioxidant capacity seems to be determined by the amount of isoflavones that accumulate [68]. In 2011, when the levels of total phenols, flavonoids, and PPGs were much lower than in the other two years, the antioxidant capacity was also lower (about 1/4). With respect to the effect of ripening, there was only a slight tendency to greater antioxidant capacity at full ripeness, S3, according to Arslan and Özcan [69].

The evolution of all these parameters for the two cultivars, two treatments, three stages of ripening, and three consecutive years is very hard to interpret. In particular, the interaction between the different variables makes the results difficult to standardize. It was therefore necessary to perform a study that would allow us to better understand these variables and their relative influence on the characteristics of the olives. To this end, we chose to use a two-step procedure–first MDS, and then a PTA. To interpret the results with this new methodological approach, it was first necessary to identify and study the similarity of the cases by way of the MDS procedure instead of the standard inter-structure analysis of PTA (without having to determine the vector correlation coefficient matrix). We could then evaluate the individual compromises by means of the PTA's compromise or consensus space, and finally analyse the variables and individuals by way of the PTA's standard intra-structure analysis.

### Similarity between years

**Fig 1:**
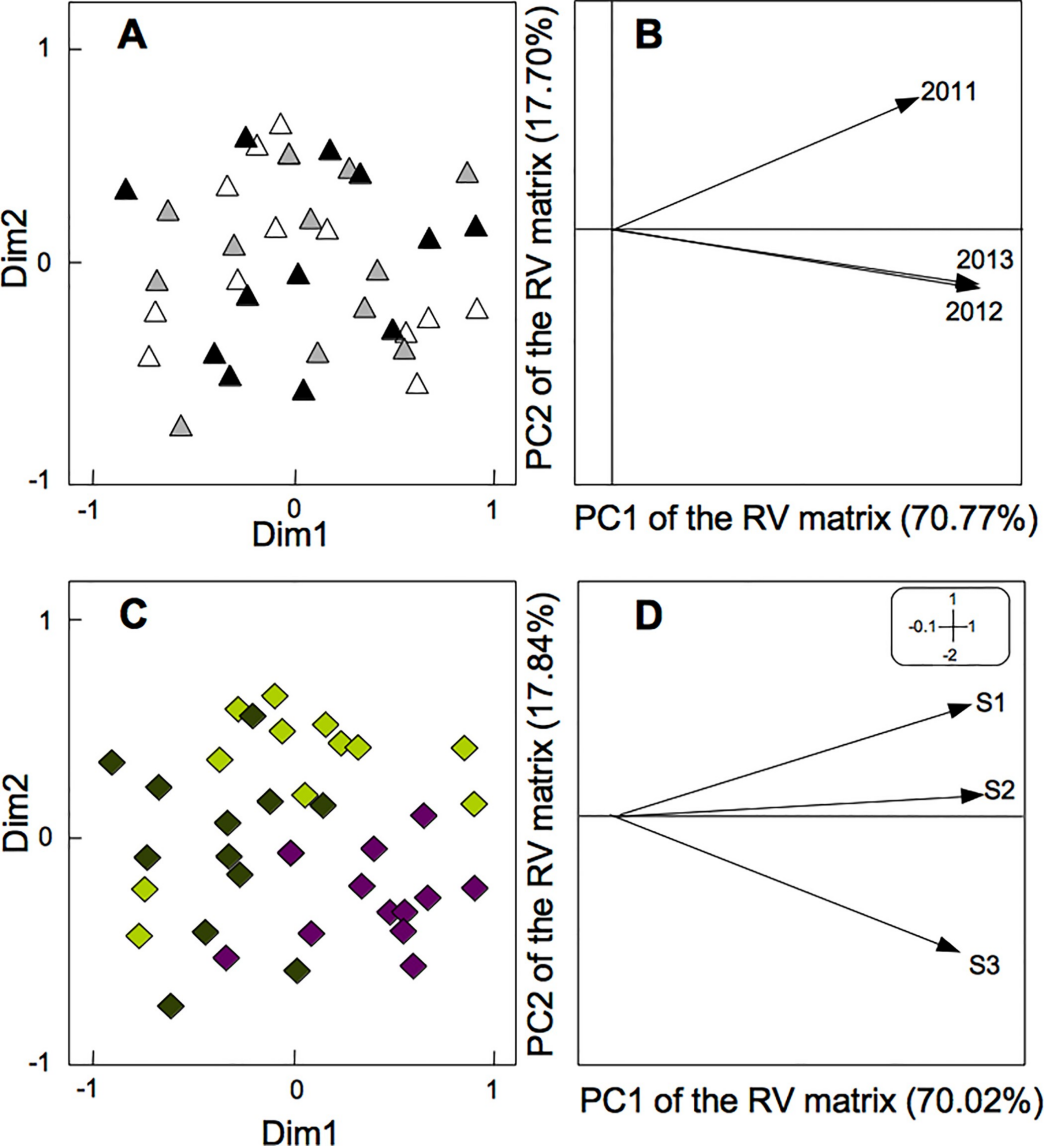
Plots of MDS common space and similarity as given by the RV coefficient analysis. **A** and **C** represent MDS, for the different years: 2011 (empty triangle), 2012 (gray triangle) and 2013 (black triangle)-, and ripening stages: S1 (light green), S2 (dark green) and S3 (purple), respectively; **B** and **D** represent RV coefficient, for the different years and ripening stages, respectively.

The MDS procedure gave a percentage of variance explained of 98.33%, and a Kruskal stress of 1.67%, implying a near perfect fit. Fig 1 shows the results of the MDS and the correlation coefficient matrix analysis used to identify the third axis of the data set. The analysis of the correlation coefficient matrices between data vectors was carried out using the ADE4 package of the R statistical computing environment. One observes (Fig 1A) that the olive samples were fairly homogeneously distributed over the common space for the different years. This agrees with the correlation coefficient matrix analysis (Fig 1B) which showed a strong common structure in 2012 and 2013, but less so in 2011. However, when the ripening stage was considered (Fig 1C), the olive samples presented a clustering trend. This was confirmed by the correlation coefficient matrix analysis (Fig 1D) since the corresponding three vectors were not close to each other. Given these results, the third axis chosen for the data array was taken to be the year (Table 3, and S1 Table).

A hierarchical cluster analysis was performed to check the differences between applying CA-PTA and MDS-PTA methods. The agglomeration schedule results showed there to be three possible clusters according to the point of formation of the elbow. In the Table 4 and in the S1 Fig, one can see that total proteins, flavonoids, NADH oxidation and POX activity significantly vary among clusters. However, in considering the clustering by year and by ripeness stage, we observed only a poor separation of individuals between clusters, unlike the MDS case (Fig 1). The proposed MDS-PTA method would thus seem to be a reasonable alternative to consider in similar cases of low grouping power.

**Table 4:** Mean values and standard desviation in the three clusters.

Parameter	Cluster 1	Cluster 2	Cluster 3
DW/FW	0.29±0.02	0.33±0.03	0.30±0.04
Soluble amino acids	2277.07±1063.93	4227.54±2025.97	2256.96±944.96
Total proteins	1.27±0.56^b^	2.52±0.77^a^	1.51±0.63^ab^
Total phenols	2796.89±879.88	3327.85±904.56	2487.95±566.3
Total flavonoids	6144.22±1518.23^b^	8102.41±1126.46^a^	5745.00±945.34^b^
Total PPGs	10097.94±2657.05	12305±2518.37	9901.03±1971.65
NADH oxidation	553.87±278.13^b^	541.82±249.2^b^	1095.51±373.3^a^
O_2_^.-^ production	250.92±98.07	279.07±129.8	445.73±215.83
SOD	92.56±50.25	100.36±55.15	146.53±46.69
POX	92.78±35.69^b^	71.83±29.03^b^	173.45±45.59^a^
PPO	467.73±168.25	402.28±55.12	334.64±126.81
FRAP	75.7±43.85	87.15±47.77	76.69±37.41

Soluble amino acids and total proteins expressed as mg g^-1^ FW; NADH oxidation, O_2_^.-^ production and POX activity expressed as nmoles min^-1^ mg^-1^ protein; SOD and PPO activities expressed as U mg^-1^ protein; total phenols, total flavonoids, total PPGS and total FRAP expressed as μg g^-1^ FW.

### Analysis of individuals

The compromise matrix is the linear combination of the matrices of scalar products representing each data table [32] that best represents the data as a whole. In the present case, the scree plot of eigenvalues showed that the first two axes accounted for 70.9% of the total statistical inertia (so-called because of its computation's formal analogy with the moment of inertia tensor in physics).

**Fig 2:**
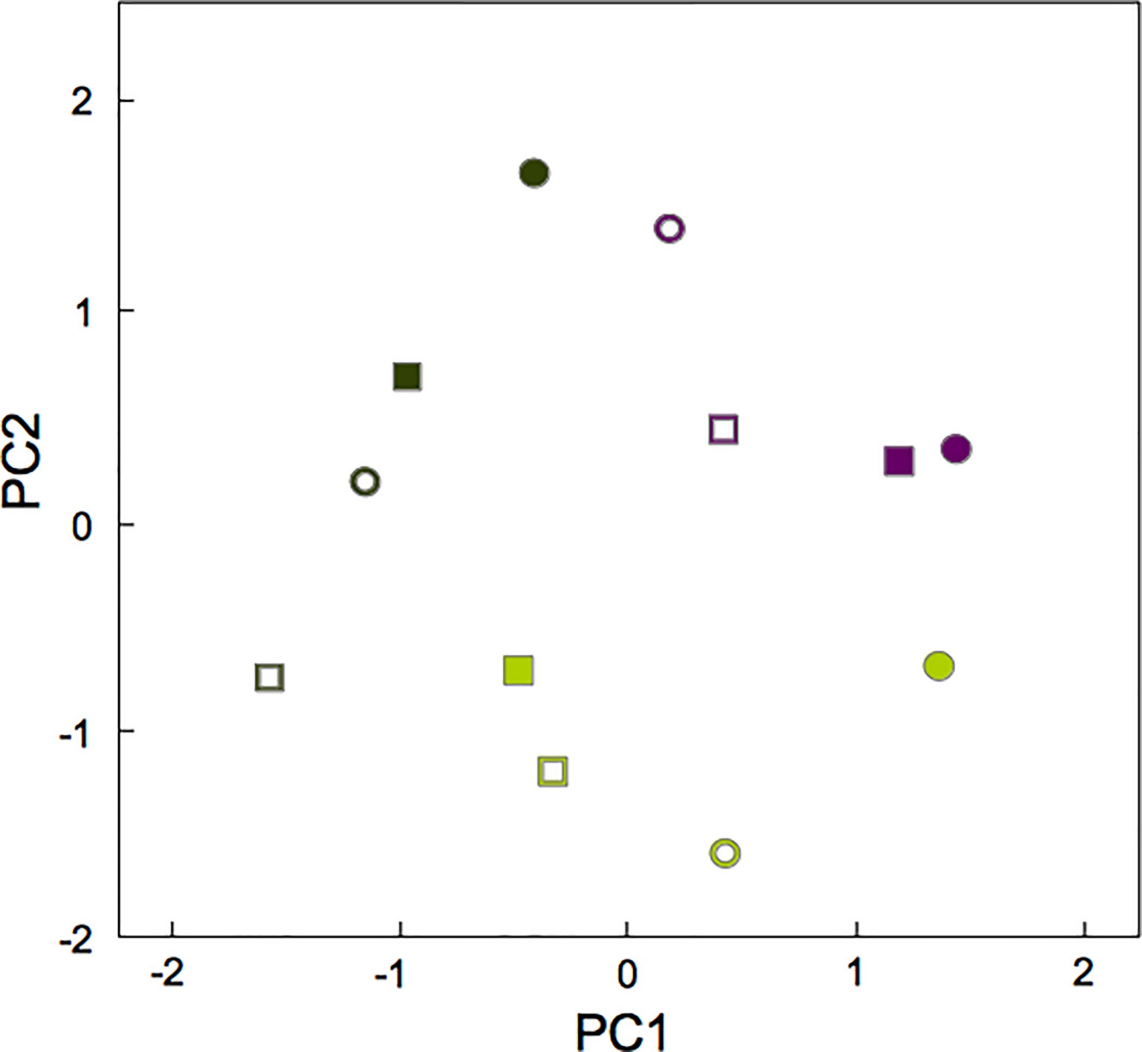
The compromise matrix–map of the first two factorial coordinates. cv. Manzanilla (squares) and Morisca (circles), rainfed (full) or irrigated (hole), respectively. The ripening stages, S1, S2 and S3 as Fig 1.

The compromise analysis reveals groupings according to ripening stage (Fig 2). This confirms that the main differences among olive samples are due to the fruits' ripeness, and is coherent with the MDS and vector correlation coefficient matrix results (Fig 1C and 1D) and with not taking the ripening stage as the third axis with which to construct the three-dimensional data array. One also observes (Fig 2) that the irrigated and rainfed regimes form sub-groups in S2 and S3.

The S1 Table shows the original data but, after compromise analysis, one can observe that the years have not influence. The significant differences of the rest of parameters and their interactions was analysed (Table 5). The ripening stage was the factor with more significant influence on oxidant and antioxidant compounds, followed by the cultivar. Total protein was the main significant differences (the lowest p-value) are linked to the ripening stage.

**Table 5:** Mean content of the chemical parameters and estimation of the influence factors (*p*-value).

Parameter	Mean	r_i_	c_j_	i_k_	r_i_·cj	r·i_k_
DW/FW	0.30±0.02			0.004		
Soluble amino acids	2866.35±1188.47	1.46·10^−5^	8.28·10^−5^			
Total proteins	1.73±0.74	1.64·10^−9^	4.64·10^−6^			
Total phenols	2856.15±428.63	3.06·10^−5^				
Total flavonoids	6609.48±971.08	0.001				
Total PPGs	10706.68±1329.36	0.032		0.007		
NADH oxidation	730.74±252.12	0.039				
O_2_^.-^ production	324.46±92.90					0.01
SOD	112.93±39.34		3.06·10^−5^			
POX	113.27±39.98	0.002				
PPO	403.37±77.18	0.042	0.016		0.009	
FRAP	79.53±10.99	0.002		0.031		

r^th^ ripening stage (green, veraison, and black or mature); c^th^ olive cultivar (Manzanilla, and Morisca); i^th^ irrigation (rainfed -NI-, and irrigated -FI-). Non-significant interaction was not shown. Soluble amino acids and total proteins expressed as mg g^-1^ FW; NADH oxidation, O_2_^.-^ production and POX activity expressed as nmoles min^-1^ mg^-1^ protein; SOD and PPO activities was expressed as U mg^-1^ protein; total phenols, total flavonoids, PPGS and total FRAP expressed as μg g^-1^ FW.

Although a MANOVA followed by a post-hoc test was determine the significance of any differences in oxidant and antioxidant activities and total phenols (Table 5), we considered it better to address this issue with the inter-structure and intra-structure analyses.

### Oxidant and antioxidant activities, and total phenols: inter-structure and intra-structure-analysis

**Fig 3:**
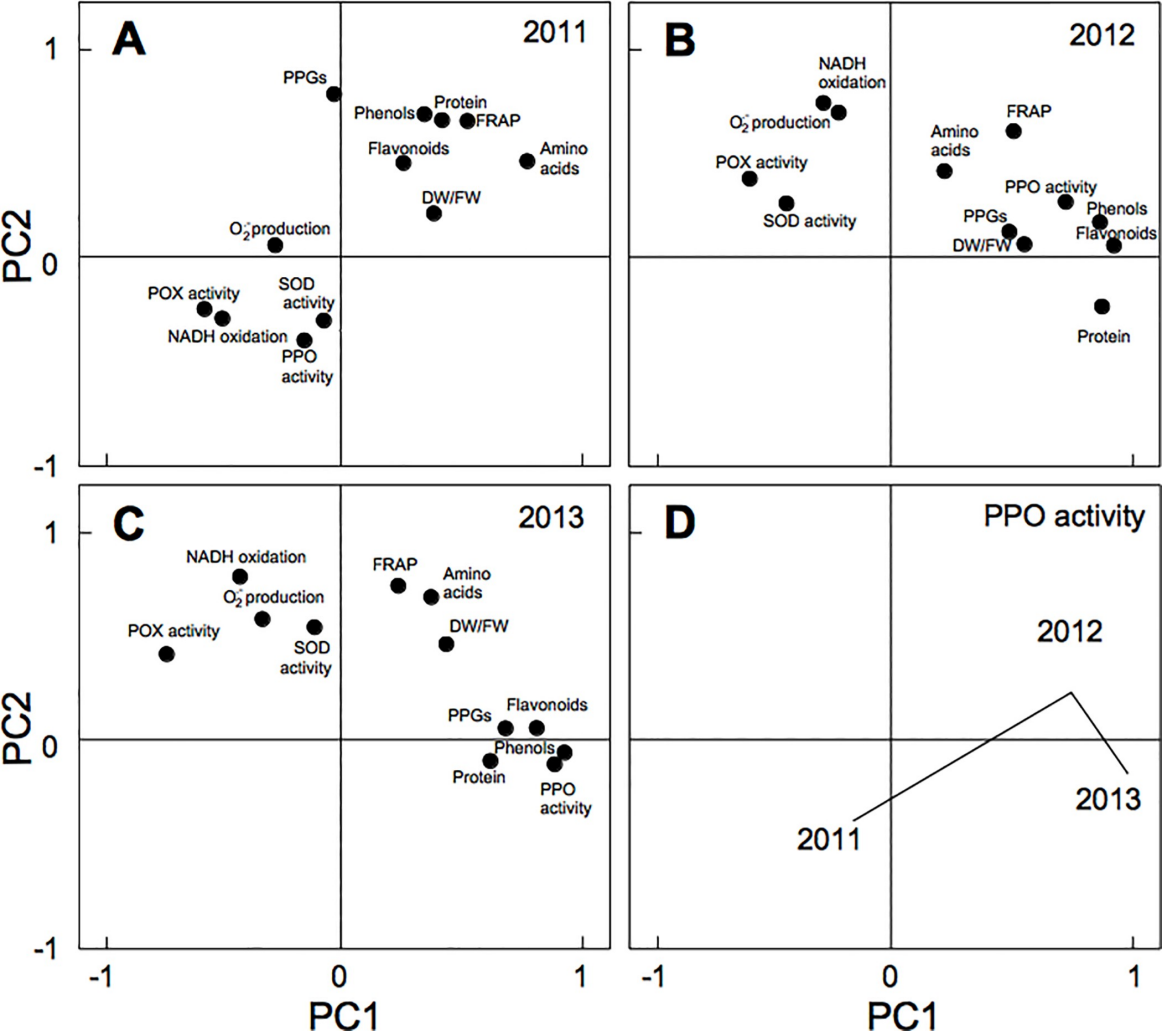
Intra-structure maps. Total phenol, PPGs and flavonoids, DW/FW, Total soluble amino acids and protein, NADH oxidation, O_2_^.-^ production, SOD, POX and PPO activities, and FRAP, for the different year, **A)** 2011, **B)** 2012 and **C)** 2013. **D)** Intrastructure map for PPO activity for 2011, 2012 and 2013.

The inter-structure analysis applies a PCA to reveal the information that is common to all the sample data [70]. A PCA applied to the inter-structure matrix (which represented 95.1% of the total inertia) clearly showed the great stability of the spatial structure. In the inter-structure analysis (S2 Fig), one observes, e.g., for phenols and PPGs, that the biochemical parameter data vary, and that they form groups especially according to ripening stage. The S3 samples particular tend to stand out, although there is no such tendency for O_2_^.-^ production, amino acids, DF/DW, or NADH oxidation. In other cases, the results obtained appear very grouped in one or more ripening stages, but not in all. At a certain moment they appear intermixed, influencing other factors such as variety or cultivation conditions. This result shows how those antioxidant components that are very closely related to ripening are grouped in response to the olives' ripeness stage, independently of the cultivation conditions. On the contrary, such enzymatic activities as NADH oxidation and O_2_^.-^ production intervene in ripening but are also indicators of processes of oxidative stress in response to biotic or abiotic stresses, and these stresses can occur at any time of development, independently of the fruits' ripening process. In this regard, the year-to-year variability of biotic and abiotic factors and stresses, and the irrigation or rainfed cultivation conditions (associated with the degree of water stress) may be more determinant than was the case for phenolic compounds. The same is the case for the antioxidant SOD and POX activities (Fig 3, and S2 Fig) which are involved in the response to oxidative stress. Since they do not depend just on the stage of ripeness, they are less clearly grouped in terms of that variable. In particular, olives grown under irrigation have different characteristics from those grown under rainfed conditions in regard to their antioxidant response to the oxidative stress induced by water stress.

The intra-structure analysis showed 2011 to be better represented by PC2, but 2012 and 2013 by PC1, and that the biochemical parameters form clusters more clearly in 2012 and 2013 than in 2011 (Fig 3A, 3B and 3C). In all cases, the enzymatic oxidant and antioxidant activities appeared to be closely linked, as also (in another cluster) were the phenols, flavonoids, PPGs, and FRAP. This clustering was repeated in all three cases, with the years 2012 and 2013 being particularly similar to each other.

The intra-structure analysis also allows one to visualize how the parameters change from year to year (sometimes called trajectory analysis). For instance, PPO (Fig 3D) is in the third quadrant in 2011, in the first quadrant in 2012, and in the fourth quadrant in 2013. The length of the path between 2011 and 2012 reflects a large difference in the mean values, while the relative shortness of the path between 2012 and 2013 reflects the similarity of the values in these two years. In contrast, the FRAP and amino acid values remained stable in the first quadrant. The use of this approach has allowed us to analyse a large number of variables that interdependently affect the final quality of the olives, making it possible to identify the factors (cultivar, irrigation, year, ripeness) that most influence the olive's oxidant and antioxidant systems, soluble amino acids, FW and DW, and FRAP. Our results provide an easily interpreted, overall view in consonance with the work of Gourdol et al. [29] and Darviche-Criado et al. [31] who applied this methodological procedure to the influence of multiple variables on water quality.

Common space analysis is a good approximation to vector correlation coefficient matrix analysis. Our proposal allows the third factor behind the data to be identified, something that is far from straightforward with cluster analysis. It also allows a very large volume of data to be organized. In particular, this methodological approach allows the most influential of a group of factors to be identified, regardless of any interactions among them. Thus, in the present case, it was the ripeness stage of the olives that was clearly discriminatory, much more so than the cultivar, irrigation, or the year. This analysis allows one to visualize parameters that are grouped together because of their close relationship with physiological processes–in this case, ripening. Phenols and the total antioxidant capacity showed a strong grouping, being closely related in all three years studied. In some ways the case was similar with the oxidant and antioxidant activities, although now there was also an influence of the year as a factor, with these parameters depending more on highly changeable external factors. This dependence meant that there was none of the close and stable grouping that had been found in the case of the phenols.

## Supporting information

S1 Table Biochemical parameters corresponding to olive fruits harvested in 2011, 2012, and 2013 (See M&M). (DOCX)

S1 Fig Agglomeration schedule, and year and ripening stage dendrogram generated by Ward’s hierarchical agglomerative clustering method. When represented by dendograms, the clusters do not have coordinates with which to measure their degree of dissimilarity by means of a Euclidean distance. One can only say that there are some number *x* of clusters. This is not the case with MDS which indeed does allow a distance measure of dissimilarity. For this reason, it has the additional advantage of being closer than CA-PTA to the objective of PTA's inter-structure analysis. Insert agglomeration coefficient.
(TIF)

S2 Fig Inter-structure maps. **A)** Total phenols, **B)** Total flavonoids, **C)** Phenylpropanoid glycosides, **D)** DW/FW, **E)** Total soluble amino acids, **F)** Total protein, **G)** NADH oxidation, **H)** O_2_^.-^ production, **I)** SOD, **J)** POX, and **K)** PPO activities, and **L)** FRAP. cv. Manzanilla (squares) and Morisca (circles), rainfed (full) or irrigated (hole), respectively, and ripening stages: S1 (light green), S2 (dark green) and S3 (purple).
(TIF)
