# Supplementary material for: Influence of cultivar, irrigation, ripening stage, and annual variability on the oxidant/antioxidant systems of olives as determined by MDS-PTA
Source: PLoS One. 2019 Apr 18;14(4):e0215540. doi: 10.1371/journal.pone.0215540 (PMC6472774; doi:10.1371/journal.pone.0215540)
Supplement: S1 Table — (DOCX) [file pone.0215540.s001.docx]

**S1 Table.** Biochemical parameters corresponding to olive fruits harvested in 2011, 2012, and 2013 (See M&M).

| **Year** | **cv** | **Irrigation** | **IM** | **DW/FW** | **Soluble amino acids** | | **Total**  **proteins** | **Total**  **phenols** | **Total**  **flavonoids** | **Total**  **PPGs** | **NADH oxidation** | **O_2_^.-^ production** | **SOD** | **POX** | **PPO** | **FRAP** |
| --- | --- | --- | --- | --- | --- | --- | --- | --- | --- | --- | --- | --- | --- | --- | --- | --- |
| **2011** | **MOR** | **NI** | **S1** | 0.264±0.022 | | 3321.6±301.5 | 0.963±0.100 | 1671.2±55.4 | 4356.5±324.6 | 7269.5±513.0 | 354.9±84.6 | 309.9±59.9 | 42.5±9.9 | 60.2±4.9 | 1080.1±136.8 | 21.7±0.8 |
|  |  |  | **S2** | 0.286±0.030 | | 3503.5±331.0 | 1.271±0.040 | 1719.1±261.0 | 4284.2±497.0 | 6942.7±191.9 | 596.4±160.5 | 142.7±59.7 | 50.8±9.0 | 161.8±35.5 | 298.8±33.7 | 26.7±0.6 |
|  |  |  | **S3** | 0.347±0.050 | | 6055.7±65.4 | 3.380±0.400 | 2213.6±39.3 | 6874.6±181.1 | 9305.7±1787.9 | 422.0±137.0 | 207.6±61.0 | 43.9±7.3 | 60.8±13.0 | 379.9±70.1 | 27.9±0.4 |
|  |  | **FI** | **S1** | 0.300±0.020 | | 2761.2±430.7 | 1.009±0.090 | 1746.0±51.6 | 5171.0±146.6 | 7761.5±550.8 | 248.9±25.2 | 148.0±33.5 | 44.9±9.5 | 32.6±4.7 | 1026.5±193.9 | 21.5±0.9 |
|  |  |  | **S2** | 0.265±0.030 | | 2319.7±409.0 | 1.150±0.200 | 1726.1±12.3 | 4642.4±529.3 | 6215.9±498.7 | 452.5±22.4 | 158.9±25.2 | 49.3±7.9 | 101.8±9.6 | 718.4±75.2 | 26.5±0.6 |
|  |  |  | **S3** | 0.318±0.020 | | 5423.7±204.0 | 3.750±0.500 | 2221.6±14.1 | 7023.2±65.8 | 9894.0±860.7 | 506.7±51.0 | 280.1±81.8 | 58.4±17.9 | 98.0±21.0 | 336.3±29.8 | 28.0±0.3 |
|  | **MAN** | **NI** | **S1** | 0.351±0.033 | | 2087.0±324.0 | 0.652±0.130 | 1680.7±73.2 | 4448.4±331.5 | 7162.5±423.5 | 904.8±130.8 | 393.2±50.3 | 140.7±18.5 | 187.4±17.1 | 666.7±105.5 | 22.0±1.0 |
|  |  |  | **S2** | 0.289±0.010 | | 1015.8±149.0 | 1.067±0.160 | 2002.5±75.6 | 5230.3±812.5 | 7883.4±724.2 | 688.7±43.5 | 355.7±23.7 | 58.5±10.4 | 124.7±9.6 | 349.7±34.1 | 26.6±0.6 |
|  |  |  | **S3** | 0.362±0.030 | | 3962.7±73.8 | 3.360±0.300 | 2217.9±33.7 | 6997.7±174.5 | 10285.0±944.7 | 252.7±19.0 | 61.2±17.0 | 66.3±14.5 | 51.6±5.2­­ | 260.2±66.7 | 27.9±0.3 |
|  |  | **FI** | **S1** | 0.324±0.030 | | 2513.2±324.7 | 0.565±0.060 | 1791.6±44.6 | 5347.1±115.4 | 8186.3±322.1 | 875.9±153.1 | 392.8±52.5 | 135.8±14.3 | 162.5±12.2 | 650.8±49.1 | 22.0±0.8 |
|  |  |  | **S2** | 0.289±0.020 | | 1115.5±185.3 | 1.191±0.170 | 1958.2±168.3 | 5152.3±817.0 | 6291.6±816.9 | 745.3±135.5 | 202.7±17.5 | 49.4±11.3 | 99.9±24.2 | 462.7±76.9 | 26.8±0.7 |
|  |  |  | **S3** | 0.364±0.061 | | 4332.5±187.0 | 3.070±0.500 | 2226.2±23.7 | 6966.5±141.7 | 10275.2±671.9 | 541.6±77.9 | 225.8±87.0 | 64.9±15.8 | 79.3±10.0 | 506.4±72.3 | 27.8±0.1 |
| **2012** | **MOR** | **NI** | **S1** | 0.380±0.006 | | 2441.6±272.0 | 1.860±0.170 | 3984.4±574.6 | 10430.4±1048.0 | 16208.4±1217.2 | 444.1±46.9 | 258.6±66.8 | 67.5±6.9 | 1096.9±20.8 | 479.5±37.2 | 100±37.9 |
|  |  |  | **S2** | 0.318±0.020 | | 2453.8±271.0 | 2.189±0.300 | 2987.5±422.0 | 6904.1±657.6 | 11998.3±1274.0 | 2079.7±127.0 | 1053.7±248.0 | 120.2±10.0 | 261.0±19.1 | 424.6±110.0 | 98.2±6.9 |
|  |  |  | **S3** | 0.337±0.020 | | 5239.6±907.9 | 2.545±0.100 | 4240.2±758.9 | 9237.1±1549.2 | 13571.0±1867.7 | 902.6±243.0 | 433.3±70.0 | 104.2±10.7 | 161.9±28.8 | 368.0±45.9 | 124.4±7.4 |
|  |  | **FI** | **S1** | 0.295±0.010 | | 1592.0±277.2 | 1.557±0.180 | 3384.1±456.9 | 7839.6±976.4 | 14300.1±1051.8 | 478.4±64.7 | 497.7±48.4 | 60.6±0.5 | 153.4±18.6 | 351.8±57.6 | 97.0±5.7 |
|  |  |  | **S2** | 0.259±0.030 | | 4249.3±514.0 | 2.292±0.100 | 2049.3±171.0 | 4875.0±479.0 | 7095.4±438.4 | 1144.0±388.0 | 638.9±169.0 | 90.5±17.0 | 255.1±30.4 | 289.7±11.2 | 98.7±11.3 |
|  |  |  | **S3** | 0.300±0.008 | | 5523.0±855.2 | 2.505±0.100 | 3759.8±1153.1 | 8462.3±1600.0 | 10050.2±1765.9 | 754.6±239.0 | 531.3±131.2 | 92.8±12.4 | 100.7±16.6 | 304.8±32.5 | 110.8±18.3 |
|  | **MAN** | **NI** | **S1** | 0.324±0.015 | | 1428.7±399.6 | 0.898±0.160 | 3263.9±364.8 | 7960.9±996.7 | 13187.8±1421.3 | 787.5±73.4 | 492.6±70.2 | 162.1±14.1 | 205.7±31.4 | 352.9±63.3 | 127.5±11.5 |
|  |  |  | **S2** | 0.341±0.008 | | 1598.5±53.6 | 0.982±0.070 | 1999.6±307.0 | 5568.6±338.9 | 10955.1±1362.5 | 1034.7±205.0 | 427.2±101.0 | 226.0±5.7 | 143.3±28.0 | 296.6±57.5 | 118.6±9.6 |
|  |  |  | **S3** | 0.292±0.014 | | 2538.7±728.3 | 1.960±0.170 | 4077.1±327.6 | 8799.3±860.6 | 13867.0±1381.2 | 916.7±206.8 | 381.8±51.3 | 141.6±15.7 | 99.9±19.2 | 350.9±39.0 | 125.4±12.1 |
|  |  | **FI** | **S1** | 0.303±0.010 | | 2484.3±239.5 | 0.759±0.210 | 2920.6±683.5 | 5776.8±517.5 | 10998.5±1122.3 | 390.7±67.5 | 319.6±52.3 | 171.7±29.6 | 167.3±38.7 | 341.3±31.9 | 103.0±10.6 |
|  |  |  | **S2** | 0.262±0.020 | | 3185.0±298.0 | 1.292±0.100 | 2257.7±193.0 | 5178.1±320.9 | 10468.1±971.9 | 955.1±60.0 | 327.3±79.0 | 187.1±37.0 | 160.7±15.0 | 276.4±32.5 | 97.7±5.7 |
|  |  |  | **S3** | 0.278±0.015 | | 2541.7±234.2 | 2.160±0.380 | 4323.5±362.1 | 7895.0±673.1 | 10892.1±1663.6 | 856.8±102.8 | 341.1±49.6 | 155.9±20.9 | 73.6±23.8 | 475.5±49.1 | 135.9±16.3 |
| **2013** | **MOR** | **NI** | **S1** | 0.290±0.022 | | 1843.6±258.1 | 1.449±0.210 | 3825.9±249.8 | 8901.0±438.4 | 15009.9±860.3 | 386-1±121.8 | 251.9±29.7 | 41.9±7.9 | 77.1±9.1 | 455.3±49.9 | 109.0±21.1 |
|  |  |  | **S2** | 0.312±0.020 | | 2296.7±307.5 | 2.058±0.280 | 3369.4±546.4 | 8444.3±755.2 | 12134.0±602.1 | 1146.8±183.8 | 333.6±72.1 | 128.3±14.6 | 146.9±36.0 | 280.2±23.6 | 92.6±15.8 |
|  |  |  | **S3** | 0.316±0.019 | | 2102.0±150.1 | 1.947±0.210 | 3398.9±142.2 | 7067.3±760.0 | 11705.5±1567.7 | 186.9±29.3 | 104.1±34.8 | 129.6±14.9 | 21.1±1.9 | 354.6±56.8 | 106.1±14.0 |
|  |  | **FI** | **S1** | 0.270±0.017 | | 1604.6±180.6 | 1.366±0.150 | 3673.2±418.6 | 5539.1±231.8 | 13527.9±1271.7 | 324.5±25.7 | 252.1±50.2 | 50.7±7.8 | 56.2±8.1 | 424.1±35.2 | 98.9±11.1 |
|  |  |  | **S2** | 0.296±0.020 | | 2125.5±330.9 | 2.198±0.210 | 2854.5±412.0 | 5629.5±983.5 | 13134.6±424.2 | 677.9±107.4 | 273.3±25.1 | 105.2±10.0 | 165.5±5.7 | 250.6±56.3 | 69.1±8.8 |
|  |  |  | **S3** | 0.274±0.017 | | 3153.5±298.9 | 2.013±0.260 | 3230.6±97.7 | 6182.8±301.8 | 9332.2±956.5 | 1206.0±191.0 | 432.0±66.1 | 109.6±18.7 | 158.4±15.8 | 306.8±45.3 | 85.1±5.9 |
|  | **MAN** | **NI** | **S1** | 0.309±0.028 | | 1228.7±146.3 | 0.628±0.090 | 2937.2±389.1 | 5477.6±251.2 | 10760.8±961.7 | 308.1±37.5 | 294.3±58.1 | 114.0±17.5 | 52.4±11.2 | 249.0±54.3 | 91.6±11.3 |
|  |  |  | **S2** | 0.299±0.030 | | 1778.5±281.6 | 1.487±0.080 | 2817.6±140.0 | 6252.6±781.0 | 12566.9±1014.4 | 1272.4±225.0 | 358.8±74.9 | 208.1±15.0 | 180.6±32.2 | 215.2±18.7 | 115.9±17.5 |
|  |  |  | **S3** | 0.317±0.029 | | 1977.7±277.3 | 1.848±0.250 | 3598.9±108.7 | 6849.0±291.3 | 11899.8±172.7 | 624.5±50.2 | 240.3±45.9 | 213.0±33.9 | 71.6±10.9 | 313.8±61.9 | 120.5±20.3 |
|  |  | **FI** | **S1** | 0.252±0.006 | | 1139.1±216.4 | 0.576±0.140 | 3347.7±638.9 | 6447.8±569.1 | 12304.3±1111.2 | 345.5±74.0 | 238.9±32.0 | 110.7±18.0 | 46.2±12.3 | 258.0±38.6 | 108.7±8.3 |
|  |  |  | **S2** | 0.228±0.020 | | 975.9±186.6 | 1.266±0.240 | 2687.5±98.0 | 4739.0±359.0 | 9189.6±946.9 | 749.1±182.0 | 272.4±50.4 | 142.3±12.8 | 177.9±21.3 | 182.7±48.8 | 66.7±14.2 |
|  |  |  | **S3** | 0.285±0.015 | | 216.4±273.5 | 2.077±0.160 | 3296.3±67.9 | 6218.9±265.7 | 8791.5±565.6 | 717.6±115.0 | 219.6±65.0 | 195.9±11.3 | 65.7±13.6 | 323.9±56.8 | 95.7±17.3 |

Soluble amino acids and total proteins expressed as mg g^-1^ FW; NADH oxidation, O_2_^.-^ production and POX activity expressed as nmoles min^-1^ mg^-1^ protein; SOD and PPO activities was expressed as U mg^-1^ protein; total phenols, total flavonoids, PPGS and total FRAP expressed as µg g^-1^ FW.
