# Supplementary figures and images for: Influence of cultivar, irrigation, ripening stage, and annual variability on the oxidant/antioxidant systems of olives as determined by MDS-PTA
Source: PLoS One. 2019 Apr 18;14(4):e0215540. doi: 10.1371/journal.pone.0215540 (PMC6472774; doi:10.1371/journal.pone.0215540)

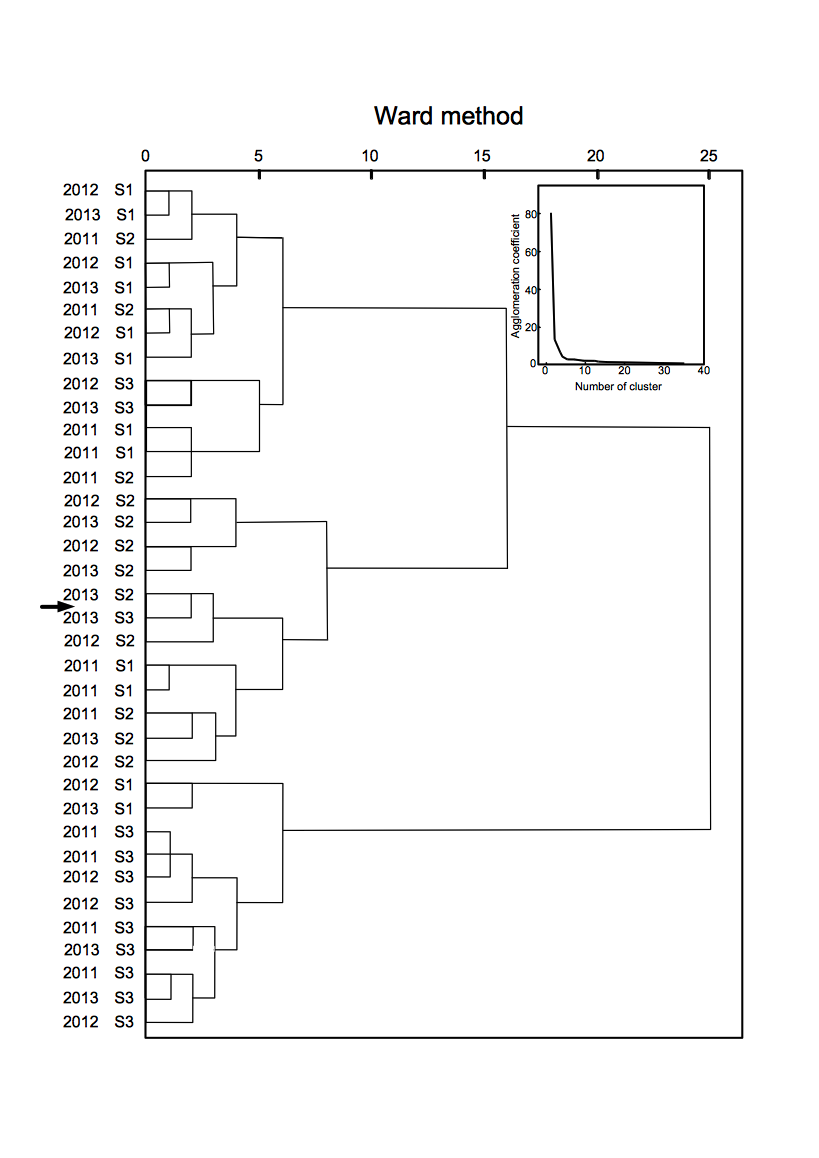

Supplement: S1 Fig — When represented by dendograms, the clusters do not have coordinates with which to measure their degree of dissimilarity by means of a Euclidean distance. One can only say that there are some number x of clusters. This is not the case with MDS which indeed does allow a distance measure of dissimilarity. For this reason, it has the additional advantage of being closer than CA-PTA to the objective of PTA's inter-structure analysis. Insert agglomeration coefficient. (TIF) [file pone.0215540.s002.tif]

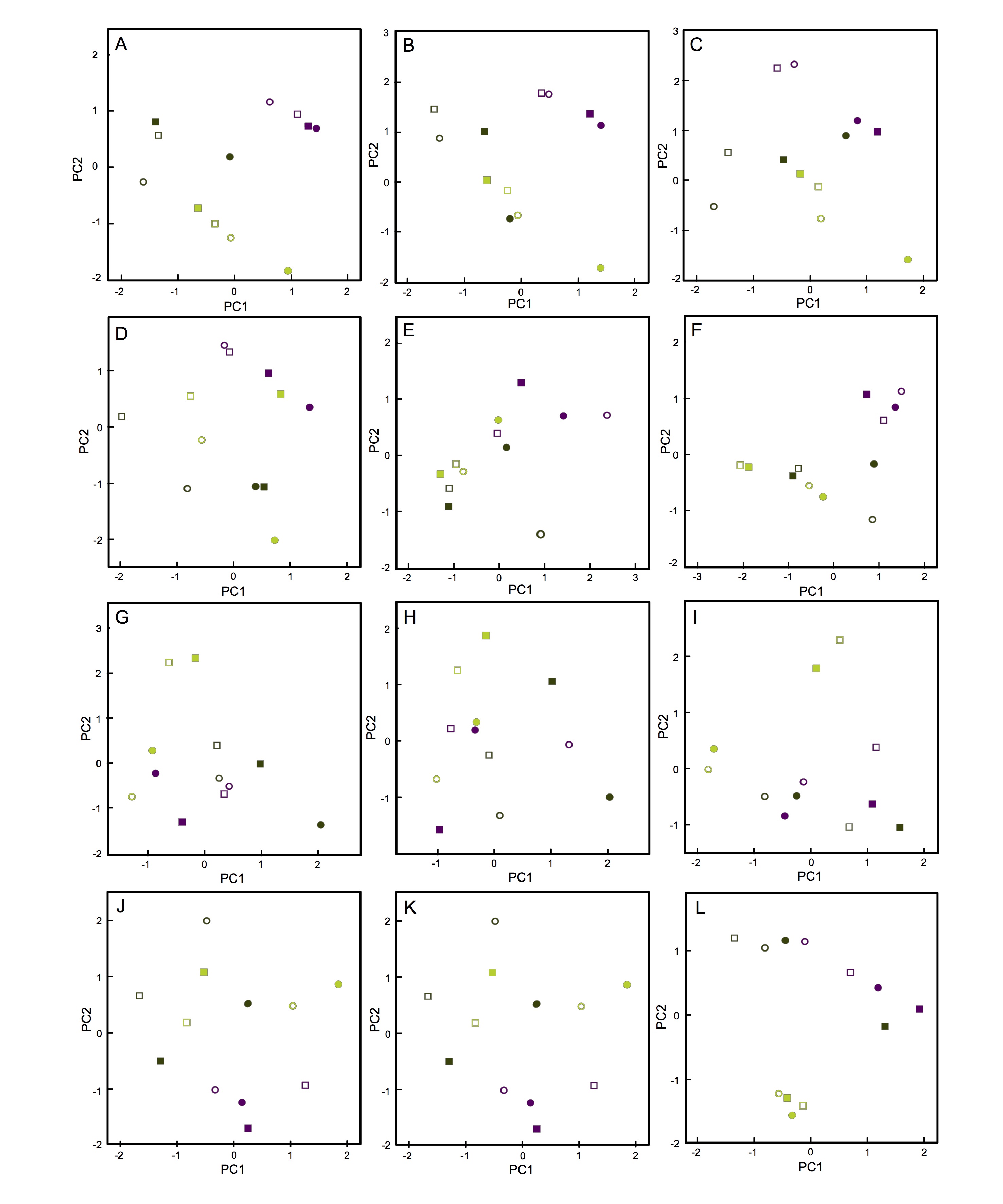

Supplement: S2 Fig — A) Total phenols, B) Total flavonoids, C) Phenylpropanoid glycosides, D) DW/FW, E) Total soluble amino acids, F) Total protein, G) NADH oxidation, H) O2.- production, I) SOD, J) POX, and K) PPO activities, and L) FRAP. cv. Manzanilla (squares) and Morisca (circles), rainfed (full) or irrigated (hole), respectively, and ripening stages: S1 (light green), S2 (dark green) and S3 (purple). (TIF) [file pone.0215540.s003.tif]
